# Exploring and mapping the universe of evolutionary graphs identifies structural properties affecting fixation probability and time

**DOI:** 10.1038/s42003-019-0374-x

**Published:** 2019-04-23

**Authors:** Marius Möller, Laura Hindersin, Arne Traulsen

**Affiliations:** 1 Department of Evolutionary Theory, Max Planck Institute for Evolutionary Biology, D-24306 Plön, Germany; 2 Complex Systems and Networks Research Group, School of Mathematical Sciences, Queen Mary University of London, Mile End Road, London, E1 4NS UK

**Keywords:** Theoretical ecology, Computational models, Evolutionary theory

## Abstract

Population structure can be modeled by evolutionary graphs, which can have a substantial influence on the fate of mutants. Individuals are located on the nodes of these graphs, competing to take over the graph via the links. Applications for this framework range from the ecology of river systems and cancer initiation in colonic crypts to biotechnological search for optimal mutations. In all these applications, both the probability of fixation and the associated time are of interest. We study this problem for all undirected and unweighted graphs up to a certain size. We devise a genetic algorithm to find graphs with high or low fixation probability and short or long fixation time and study their structure searching for common themes. Our work unravels structural properties that maximize or minimize fixation probability and time, which allows us to contribute to a first map of the universe of evolutionary graphs.

## Abstract (web summary)

Marius Möller, Laura Hindersin & Arne Traulsen develop a genetic algorithm to find evolutionary graphs with varying fixation probability and time. They identify structural properties that maximize or minimize both of these parameters and provide a first map of the universe of evolutionary graphs.

## Introduction

How does population structure affect evolutionary dynamics? This question is at the center of evolutionary graph theory, since its introduction by Lieberman et al.^1^ and remains a flourishing research topic^2–13^. While the bulk of the associated work is theoretical at this stage, there are many potential applications: In ecology, river systems often have tree-like structures and the spread of new ecotypes or species happens along the branches of this tree. However, the direct applicability of evolutionary graph theory to this ecological context is challenging, as there are many additional biological factors that could overrule the importance of population structure^14^. Another possible application is cancer initiation, where the population structure of colonic crypts can be a decisive factor in the accumulation of cancerous mutations^15^. However, it is unclear if insights from evolutionary graph theory can be directly transferred to such systems, as the dynamics of cell divisions in these crypts usually allows for more flexibility than the assumptions that need to be made to analyze evolutionary graphs and as even basic issues such as the distribution of fitness effects are often not known^16,17^. A straightforward biotechnical application would be the design of networks that maximize the chance that advantageous mutations take over a population in experimental evolution or biotechnological applications^12^. However, in that context, it is not only important if a mutant type would take over a population, but also how long this would take^18^. This is exactly the question we address here: Which costs do we need to pay in terms of the time to fixation if we maximize the probability of fixation?

**Fig. 1:**
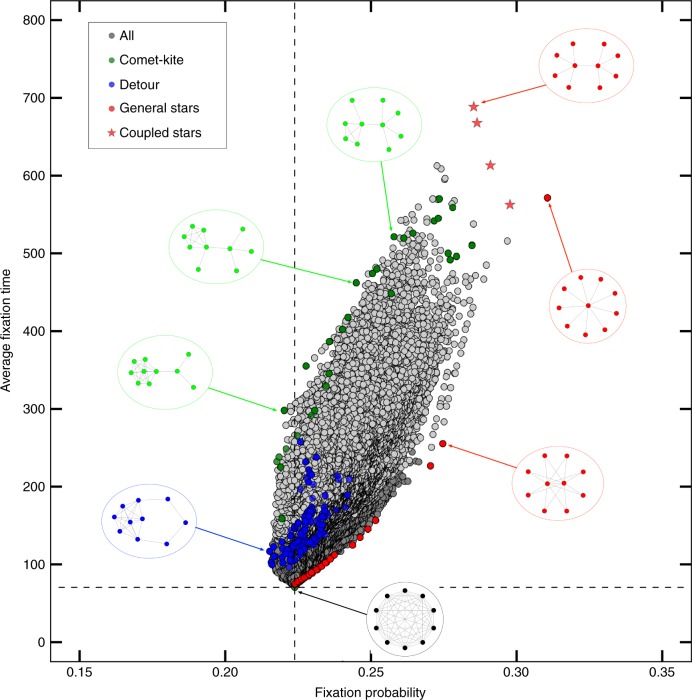
All graphs of size 10. Overview showing the fixation probability and mean conditional fixation time of all 11,716,571 graphs with 10 nodes as gray dots for *r* = 1.25 and Birth-death updating. Certain special types of graphs are portrayed showing their structure. The dashed lines indicate the fixation probability and time for the complete graph. We highlight in color three categories of graphs: The red graphs are generalized star graphs that provide high fixation probability to the mutants. A subcategory of these generalized stars are coupled stars with the highest fixation times (star symbols). The blue graphs are “detour” graphs that tend to have low fixation probability and time. The green “comet-kite” graphs represent part of the left border of the set of all graphs. They minimize fixation probability while spanning a large range of fixation time values. All other graphs outside these three categories are gray: Light gray shows those graphs that have a least one node with only a single neighbor, dark gray shows all remaining graphs

Imagine a graph of *N* nodes, connected by links between them (see Fig. 1 for some examples). In each of the nodes, there is a single individual. We will focus on two types only, a mutant and a resident. Mutants are assigned a relative fitness *r* compared to the resident’s fitness 1. In every time step, an individual is chosen for reproduction and its offspring replaces another random individual chosen for death.

More formally, we use the Moran process as a model for the invasion of a mutant type into a resident population^19^. In each time step, a single individual is selected to produce one identical offspring individual. The offspring replaces another individual, such that the population size remains constant. In the case with population structure, the individuals of the population are located on the nodes of the graph^1^. Different from the standard non-spatial Moran process, replacement is not taking place among the whole population, but only among the neighbors of the reproducing individual^1,2,4,20,21^. Different updating mechanisms are of interest, as they can strongly influence the results^11^: In Birth-death updating (Bd), a random individual is chosen for reproduction with probability proportional to its fitness to produce an identical offspring, which then replaces a random neighbor. This is sometimes referred to as “invasion process”^22^. In death-Birth updating (dB), a random individual dies and this vacant site is immediately filled by the offspring of one of its neighbors chosen with a probability proportional to their fitness. This is similar to the voter model^23^. There are many more possible updating mechanisms, e.g. bD and Db, where fitness is attached to the death step, and it has been shown repeatedly that the details of this implementation can have a major influence on the dynamics of the process^11,16,21,22,24–26^. Here we are focussing on Bd updating as the most popular version.

The complete graph, where every node is connected to every other node, corresponds to the well-mixed population that is the basis for the original Moran process^19^. It serves as a reference case to which other graphs can be compared with respect to their fixation probability and time. Amplifiers and suppressors of selection are graphs that differ from the complete graph in their fixation probability in a particular way^1,4^: An amplifier of selection in the most strict sense is a graph, where an advantageous mutant (*r* > 1) has a higher fixation probability and a disadvantageous mutant (*r* < 1) has a lower fixation probability than on the complete graph. A suppressor of selection is the reverse.

For weighted graphs, where the dynamics of reproduction is also affected by properties of the links, it has been shown that graphs that do not differ too much from the complete graph, also have a similar fixation probability^7^. More recently, it has been shown that some directed graphs can be strong amplifiers of selection^12^. However, strong amplification typically comes at a cost: Many amplifiers of selection have been found to slow down the fixation process by increasing the time to fixation^18,27–29^. For example, Frean et al. showed that the average conditional fixation time is much higher on the star than on the complete graph^18^. The simplest case are graphs such as the cycle, where all nodes form a ring such that the graph is degree homogeneous. While such graphs have the same fixation probability as the complete graph, their fixation time is increased. Overall, there exist some quite general results for the fixation probability, but the fixation time depends on the graph structure in a very subtle way and removing a link can either increase or decrease the fixation time^28^.

## Results

### An exhaustive analysis is possible only for small graphs

In ref. ^11^, it was shown numerically that most undirected random graphs, where each possible link is present with the same probability, are amplifiers of selection for Bd updating and uniform placement of the initial mutant (up to size *N* = 14). For each *N*, a large number of Erdös–Rényi graphs^30^ were generated and their fixation probability was calculated. Erdös–Rényi graphs are generated by giving every potential link the same probability to be created. Using improved numerical methods^31^, it is possible to study *all* graphs up to a certain size instead of looking at the subset of random graphs and to classify them in terms of fixation probability and time.

However, the space of all graphs increases rapidly, therefore different approaches are necessary to search through it. Starting from size *N* = 11 (more than 1 billion connected undirected graphs^32^), we employ a genetic algorithm to optimize for certain properties. The genetic algorithm is based on a small group of graphs that are screened e.g. for a high fixation probability given a certain mutant fitness *r*. Those graphs that lead to the highest fixation probability are maintained in the group, while those with small fixation probabilities are deleted. They are replaced by additional graphs which maintain some of the structural properties of the graphs with high fixation probability. This new group is screened again to filter the graphs that maximize fixation probability, and so on. This approach allows us to infer if the graph features suggested to optimize e.g., fixation probability are still optimal in much larger graphs, where an exhaustive analysis is no longer possible. For example, for low fixation time, we would assume that the genetic algorithm will find the complete graph, or a very similar graph with many links.

In particular, we focus on a search of those graphs with the highest and lowest fixation probability and time for a given *N* and *r*. We cannot guarantee that the genetic algorithm actually finds the global optimum. But for sizes up to *N* = 10, we confirm that the genetic algorithm finds exactly the same optima that we find by systematically scanning through all graphs. This worked for all the directions: minimizing and maximizing fixation probability and conditional fixation time. This serves as a proof-of-concept for the application of the genetic algorithm and suggests that it works reasonably well for larger graph sizes as well.

### Fixation probability and time are correlated

Modifying the graph structure to increase the probability that a mutation takes over often comes at a cost: The modification can at the same time increase the time it takes for the mutant to take over. To explore this issue, we visualize all graphs of size *N* = 10 in the plane of fixation probability and mean conditional fixation time for particular fitness values. Figure 1 shows that probability and time are highly correlated. For example, in biotechnology one could be interested in designing a system that either amplifies or suppresses advantageous mutants. This could be achieved by graphs that have a high fixation probability and a low fixation time or vice versa. In this context, the correlation between probability and time can be seen as a kind of tradeoff.

**Fig. 2:**
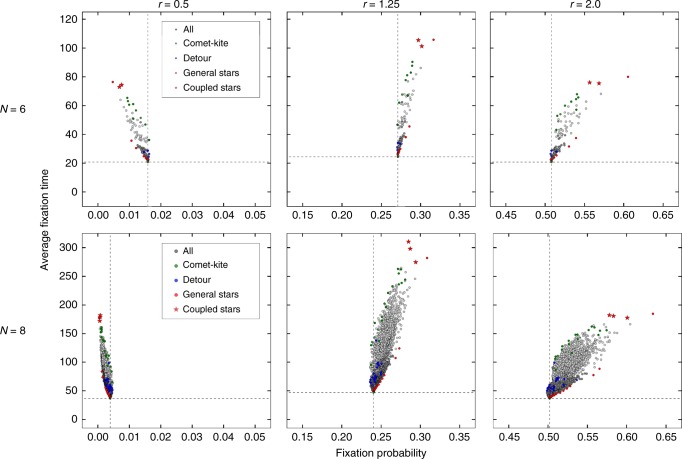
Changing graph properties with changing fitness. Overview showing the fixation probability and mean conditional fixation time of all graphs with 6 nodes (in total 112 graphs) and 8 nodes (11,117 graphs) as gray dots for different fitness values. The dashed lines indicate the fixation probability and time for the complete graph. Special graph categories are again shown in colors (cf. Fig. 1.) Light gray shows those graphs that have a least one node with only a single neighbor, dark gray shows all remaining graphs. It turns out that this distinction can capture the separation of all graphs into two regions to some extent. In the Supplementary Movie, we show an animated.gif of this figure for changing *r*

In addition, Fig. 1 highlights special categories of graphs with extremal properties, meaning it has either unusually high or unusually low values in this property. We describe in Methods how these categories are generated. Some particular graphs are of special interest: For example, compare the coupled star, which arises from coupling two smaller stars, and the generalized star with two central nodes. From a structural point of view, they only differ in that the leaves are connected to both centers instead of to only one, respectively. In terms of fixation probability, they are almost identical, but the coupled star has a much higher fixation time. As another example, the generalized star with two central nodes can also be compared to the comet-kite graphs with relatively low fixation time. They have a similar fixation time, but are very different in fixation probability. Structurally, they are also quite distinct: The comet-kite graphs have a large region of fully connected nodes, and only a few tail nodes, while the generalized star has a very small central region. The star graphs, the comet-kites and the detour graphs cover the boundaries in terms of fixation probabilities and time, but one has to keep in mind that Fig. 1 shows only one particular value of *r*. Figure 2 displays all graphs of the smaller sizes 6 and 8 in the probability-time-plane for different fitness values *r*. The graphs can be clustered in similar categories as for size 10, with changes for the extreme graphs. For disadvantageous mutants with fitness value below 1, here *r* = 0.5, the categories are roughly mirrored on the vertical line that represents the complete graph. We also provide a Supplementary Movie (as an animated.gif file) which shows how the graphs move in the plane with increasing *r*.

While we have shown special graphs that are at the boundaries in terms of fixation probability and time, one should add a note of caution: If we change the update mechanism, e.g., from selection at birth and random death (Birth-death) to random birth and subsequent local selection for birth (death-Birth), the results can be very different, see Supplementary Figure 1.

### The strongest undirected suppressors of selection

**Fig. 3:**
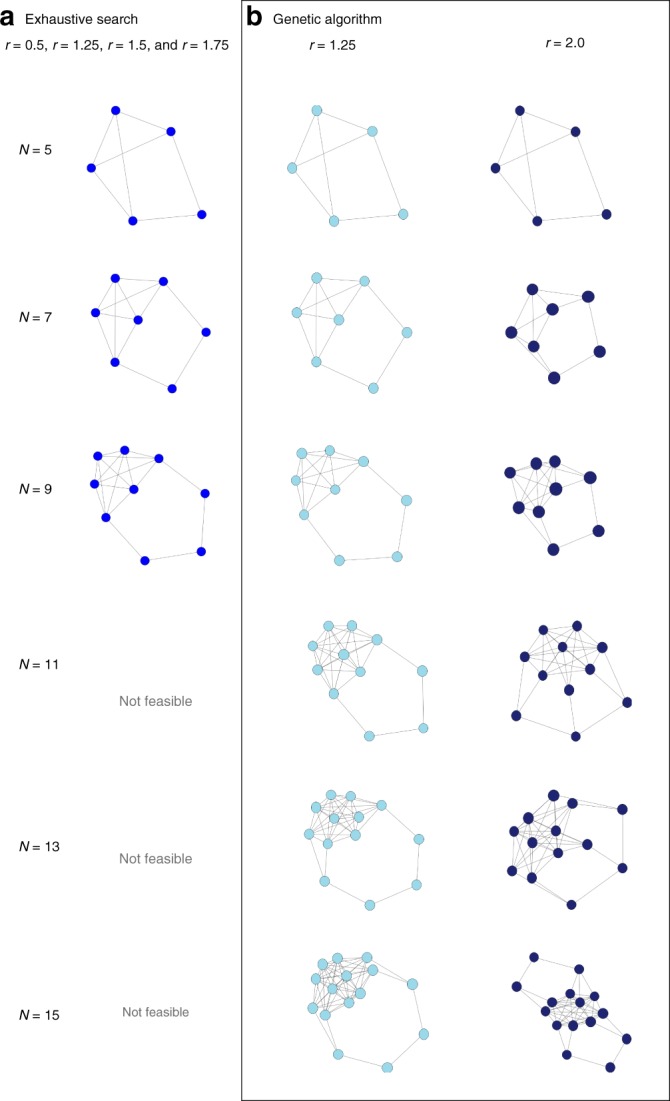
Graphs with the lowest fixation probability under Birth-death updating. **a** For small *N*, we can exhaustively search through all graphs of the respective size and find that for *r* = 0.5 (where we look for high fixation probability, because of the definition of supressors of selection), *r* = 1.25,1.5, and 1.75, the strongest suppressors are always identical, but even for *N* = 11, such a systematic search is no longer feasible, as there are more than 10^9^ undirected connected graphs (1,006,700,565). **b** For larger *N*, we thus employ a genetic algorithm to minimize the fixation probability. To reduce the probability to end up in a local minimum, we performed 5 independent runs of the genetic algorithm and took the graph with the lowest fixation probability between them. The strongest suppressors found in this way for *r* = 1.25 are – as expected – identical for *N* smaller than 9. For strong selection, here *r* = 2.0, the structures are slightly altered, as seen for *N* = 7 and *N* = 9. For larger *N*, the graphs are structurally very similar to the smaller structures depicted in (**a**), but they can still change with *r*

Let us now focus on graphs suppressing selection, i.e., reducing the fixation probability compared to the fully connected case. With our numerical algorithm, we can systematically search for the graphs with the lowest fixation probability for particular values of *r*. Studying all graphs up to size *N* = 10, we find that similar graphs are the strongest suppressors for different values of *r*. Fig. 3a shows these strongest suppressors for sizes 5 through 10. Their structure is characterized by a “core” part connected by a “detour”. They resemble a mixture of a completely connected part with a cycle part. Interestingly, their mean conditional fixation time lies between the complete graph and the cycle as well. It is also notable that for weaker selection (*r* ≈ 1) some kite-like graphs can become the strongest suppressors.

For larger population sizes, we must resort to a genetic algorithm, as an exhaustive analysis of all graphs is no longer feasible (see Methods). The genetic algorithm is based on three main steps: Competition, recombination and mutation. First, we start with fully randomly generated graphs. In the competition step, we choose those graphs with a property we want to optimize for, like highest fixation probability, as the parents of the next generation. Then, we recombine two of those parents into a new graph, and also mutate a small number of links per individual fully at random. This approach is a mix of deterministically optimizing for a property while still adding in enough randomness to make it less likely to get stuck in a local extremum. Using this genetic algorithm to search for minimal fixation probability of the graph sizes 6–10 and *r* = 1.25 leads to the same graphs as by systematically searching through all graphs, validating our approach.

For larger sizes of 11–15, similar structures remain the strongest suppressors for *r* = 1.25, but some details change. For example, at size 15 the connection between the core and the detour becomes smoother by having more intermediate nodes which belong to neither group, see Fig. 3b. Moreover, the genetic algorithm also reveals that the optimum can change with *r*. While the overall characteristic of a dense core with a detour remains important for a low fixation probability, additional detours can appear for *r* = 2.0, cf. Fig. 3b.

### The strongest undirected amplifiers of selection

We can employ the same approach and search for the highest fixation probability instead. For small *N*, a systematic search shows that the star has the highest fixation probability. For sizes of 11−16, the star is identified again as the strongest amplifier. Of course, stronger amplifiers of selection are already known^1^, but they are all directed or weighted graphs. Pavlogiannis et al. found a class of undirected graphs that they call comets which can have a higher fixation probability than stars for certain graph sizes of more than 100 nodes^33^. Comet-graphs consist of a complete graph with a star attached to one of the nodes.

### Optimizing the fixation time

Instead of focussing on the probabilities of fixation, we can also consider the time to fixation. This time is randomly distributed, with a theoretical lower bound of *N* time steps from the emergence of the mutant to fixation. We focus on the average conditional fixation time here. In our systematic search, the complete graph always emerged as the graph with fastest average time to fixation. However, the formal proof that the complete graph represents the global minimum for the average fixation time for any undirected and unweighted graph of size *N* is still an open challenge. Counterexamples exist for directed graphs^29^ and frequency dependent selection^27^.

Alternatively, we could search for the graphs that maximize the time to fixation. These are typically star-like structures, which offer many weakly connected nodes where the type of the node remains unchanged for very long time. The precise structure of this graph depends on both the size of the graph *N* and the fitness advantage *r*. For example, for *N* = 10 and *r* = 1.25, we find a coupled star as the slowest structure, cf. Fig. 1.

### The optimal graphs change with the selection strength *r*

For our numerical approach, we must naturally focus on particular values of *r* and particular graph sizes. Classifying a graph numerically as an amplifier or suppressor of selection in this way can never be a proof, as only a finite number of *r* values can be tested. In a previous numerical study, we used five values of *r*^11^ to classify graphs, as for those graphs a higher number of values for *r* did not change the results in any way. A recent paper confirms the large proportion of amplifiers with exact symbolical calculations for size 6 and 7 and numerics with small step size in *r* for sizes up to 10^34^. The vast majority of graphs falls into the following three classes of (i) regular graphs (which have the same fixation probability as the complete graph^1^), (ii) amplifiers and (iii) suppressors of selection. But some interesting exceptions exist, e.g. graphs that reduce the fixation probability for both advantageous and disadvantageous mutants^17^, and graphs that exhibit transitions from being a suppressor to being an amplifier for increasing *r*^34^. In our context, we cannot be entirely sure that the graph properties stay the same when the fitness value *r* is changed: A graph could reduce the fixation probability for certain values of *r*, but increase it for others. It turns out that there are several graphs that are neither a true amplifier nor a true suppressor of selection^34^. For example, it has been shown that the cotton-candy graph of size *N* = 10 (a kite with tail length 1) is a piecewise suppressor^35^. Cuesta et al. found a class of graphs called *l*-graphs, which are suppressors of selection for size up to *N* = 10 and for all *r*^36^. The associated mathematical proof becomes challenging for larger *N*, but it is complemented by a numerical exploration that indicates that the same result holds for *N* up to 24. These *l*-graphs are structurally similar to the graphs in Fig. 3, their “detour” is simply a link connecting two nodes that are each connected to half of the “core”.

**Fig. 4:**
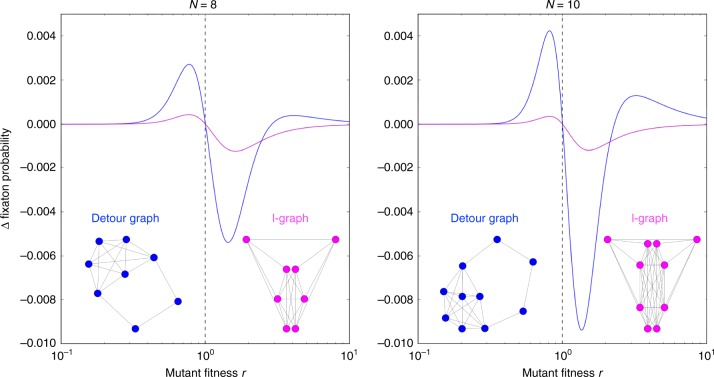
Switching between amplification and suppression with changing fitness. Up to size *N* = 10, *l*-graphs are suppressors of selection for any fitness value *r*. In contrast, detour graphs switch from suppression to amplification at a critical value of *r*. The figure shows the difference between the fixation probability from the given graphs to the complete graphs of the same size. The blue line represents the detour graph and the magenta line shows the result for the *l*-graph

Figure 4 shows that the detour graphs are actually piecewise suppressors of selection. They are very strong “suppressors” up to a certain *r* and then they transition into being an “amplifier”. We put this in quotation marks, because these graphs fulfill neither the original definition of a suppressor, nor the original definition of an amplifier. The *l*-graphs on the other hand are true suppressors of selection, their fixation probability is below the one of the complete graph for all *r* > 1 and above the complete graph for all *r* < 1^36^.

## Discussion

Any application of evolutionary graph theory will need to consider not only the probability of fixation, but also the associated average time: There is no point in using a perfect amplifier of selection that makes sure the mutations with highest fitness reach fixation when the time to achieve this increases to extreme values simultaneously. Thus, it seems to be crucial to address how fixation probability and time are related. So far, the focus has mostly been on the probability of fixation: Recently, there have been proposals to optimize graph structures to amplify selection in favour of fit mutants. Pavlogiannis et al. have shown that arbitrarily strong amplifiers of selection can be constructed from almost any graph, as long as self loops and weighted links are allowed^12^. For undirected and unweighted graphs, there is much less freedom to construct strong amplifiers. However, the simplicity of undirected and unweighted graphs allows us to ask which structural properties make a graph a strong amplifier of selection. For random graphs, it has been shown that the heterogeneity of a graph, given by the variance between the average speed at which nodes are replaced, is strongly correlated with the fixation probability^37^. In Supplementary Table 1, we show that this correlation also holds for *all* graphs of smaller size, whereas Supplementary Table 2 and Supplementary Figure 2 show that it also holds for Erdös–Rényi samples of larger graphs. To construct strong amplifiers of selection, it thus makes sense to start from graphs that are highly heterogenous.

However, despite the subtle effects of the graph structure on fixation time^28^, as we can see in Fig. 1, there is a conspicuous correlation between the fixation probability and the average fixation time. A naive optimization of fixation probability on the space of all possible undirected and unweighted graphs without self loops always yields the star (for the sizes and fitness values above 1 we studied), which also happens to be one of the slowest fixating graphs. We considered both properties at once, to discern what properties lead to a graph fixating slowly or fast, or being a strong amplifier or suppressor. In particular, we found some categories of graphs that optimize both the fixation probability and the fixation time simultaneously. Tkladec et al. call the graphs where the fixation probability cannot be increased without increasing the fixation time “Pareto-optimal”^29^, motivated by the economic idea of a Pareto optimum where no further improvement is possible without impairing another property.

When maximizing fixation probability and minimizing fixation time, we found the category of the generalized star^38^, to which the star belongs as well. However, all the other graphs in this category have a much lower fixation time than the star, making certain ones possibly better when constructing an environment where mutants with higher fitness are expected to fixate both fast and reliably.

On the other hand, when minimizing fixation probability and maximizing fixation time, we obtain the “comet-kites”. They look like kites with a few extra, comet-like tails. When maximizing fixation time alone, we obtain coupled stars instead.

Depending on which property is considered more important, a wide variety of different graphs can be optimal. Furthermore, we showed how a genetic algorithm can be used to optimize for certain properties. Not only was the genetic algorithm capable of finding the graphs for the lower sizes, which we already knew to be optimal, it could also find interesting graphs for higher sizes. This approach can be expanded to look for a combination of fixation probability and time, or in completely different directions. With the fast computational method we use for computing fixation probability and time, we are limited to sizes roughly up to *N* = 23^31^. There are many interesting findings for larger graphs, e.g., that the star is not the strongest amplifier for all sizes, but that comets can have an even higher fixation probability^33^. Our genetic algorithm can be easily combined with other methods of computation or simulation to tackle larger graph sizes.

Many open questions remain in the field of evolutionary graph theory. Our approach of looking at both the probability and the time of fixation reveals interesting graphs at the edges of optimality. With such approaches, the construction of a map of all evolutionary graphs can be envisioned.

## Methods

### Computing fixation probabilities and times

In order to compute the fixation probabilities and times, we use the adjacency matrix based approach published in ref. ^31^. Note that this approach does not exploit any symmetries of the graphs and is thus limited by the population size to *N* ≈ 23.

### Generating all graphs

To numerically generate all possible graphs up to a certain size, we use the software geng from the package “nauty”^39^. In particular, we can generate all connected, undirected graphs of a certain size with the command “geng size -c”, which can be converted to a more easily readable format with a program from the same package, showg.

The output can then be used for a shell script or a python script.

### Drawing the graphs

We use the Kamada-Kawai force-directed algorithm to plot graphs^40^. This algorithm draws graphs in a manner which clearly presents structure, we use the iGraph implementation^41^.

### Defining special graph categories

Here we describe how we define the special categories of graphs for Figs. 1 and 2. Our goal is to find those graphs that are extreme in terms of their fixation probability and time and to identify common themes in them that allow to define a whole group of graphs, see Fig. 1. For our purposes, it is easiest give a method on how to construct these graphs.

**Comet-kites**

Input parameters:

*N*: Number of nodes in the graph (integer)

*c*: Number of central, fully connected nodes (integer)

*t*: Number of comet-like tails connected to one of the central nodes (integer)

Algorithm:

1. Fully connect the first *c* nodes to generate a complete sub graph (clique) of size *c*.
2. Attach a number of *t* nodes to node number *c* in a star-like fashion.
3. Iteratively add one link respectively from every one of the remaining *N*−*c*−*t* nodes randomly somewhere to the tails.

**Fig. 5:**
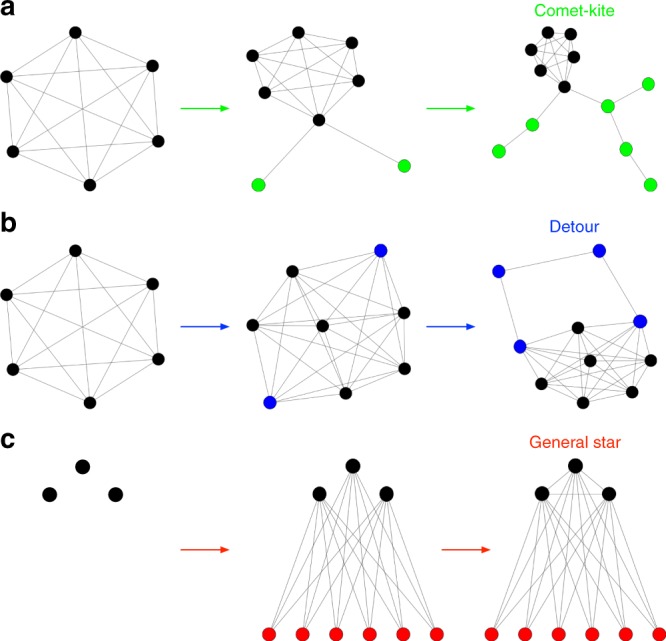
Examples for the graph generator algorithms. **a** The three steps of the comet-kite-algorithm. First the fully connected region (in black) is generated, then the “roots” of the “tails” (in green), then the random additions to the tails (also in green). **b** The three steps of the detour-algorithm. First the fully connected region (in black) is generated, then the outer region (in blue), then the detour (also in blue). **c** The steps of the general star-algorithm. First the bipartite graph (first partition shown in black) is generated, then connections between the nodes in the first partition are added

This process shown in Fig. 5a generates a single “comet-kite” graph. For the figures, we varied *c* = 1, 2, ..., *N*−1 and *t* = 1, 2 for a given *N*. Higher numbers of tails resulting in more comet-like graphs were less extreme in terms of fixation probability and time. Additionally, the standard kites are added, which are the graphs with a fully connected region and just a single line connecting to one of the nodes. This is done because it is unlikely to generate them by chance.

**Detours**

Input parameters:

*N*: Number of nodes in the graph (integer).

*c*: Number of central, fully connected nodes (integer).

Algorithm:

1. Fully connect all nodes up to node *c* to generate a complete sub graph of size *c*, which forms the inner region of the graph.
2. Generate a random integer *o* ∈ {2, min(*c*, *N* − *c* − 1)}, which is the number of outer region nodes. This range of values is chosen to avoid that the outer region is larger than the inner region and to ensure that there are enough remaining nodes for the detour.
3. Generate the detour. This means adding links from the remaining *N* − *c* − *o* nodes randomly somewhere to the outer region or other nodes in the detour until all nodes in the detour have at least 2 connections.
4. Check whether the outer region nodes are connected with the detour. If not, repeat.

This generates a single “detour” graph, the algorithm is depicted in Fig. 5b. We vary *c* from *c* = {2, 3, ..., *N* − 4}. In addition, we add the “standard” detour graphs, which are those graphs, where *o* = 2 and where the detour is connected in a ring-like fashion from one of the outer region nodes to the other.

**General stars**

Input parameters:

*N*: Number of nodes in the graph (integer).

*a*: Number of nodes in the first partition (integer).

*p*: Likelihood of the nodes in the first partition to be connected to each other (real number, 0 ≤ *p* ≤ 1). If *p* = 0, we have a standard bipartite graph. If *p* = 1, the first partition is fully connected.

1. Generate a bipartite graph with *a* nodes in one partition and *N*−*a* nodes in the other, where all nodes in one partition are connected to all nodes in the other partition.
2. Randomly connect the nodes within the first partition with probability *p*.

This generates one “generalized star” graph. We vary *a* ∈ {1, …, (*N*/2)} and *p* ∈ {0.01, 0.02, …, 1} for a given *N*.

This process is shown in Fig. 5c.

**Coupled stars**

Input parameters:

*N*: Number of nodes in the graph (integer)

*a*: Number of nodes connected to the first center of the coupled star (integer)

1. Connect the first two nodes to each other. These are the central nodes.
2. Connect *a* nodes to the first node and *N*−*a*−2 nodes to the second node.

We vary *a* ∈ {0, 1, …, (*N* − 2/2)} for a given *N*.

### The genetic algorithm

For small *N*, when looking for extreme graphs, we simply generated all graphs for a given size and then chose the ones with interesting properties. This, however, is not possible anymore for increasing sizes, since not only does the wall time per graph increase exponentially, but so does the number of graphs.

Using the Erdös–Rényi algorithm to generate a huge number of graphs and search through them, as in^11^, also does not seem promising, since, as seen in Fig. 1, the extreme graphs have very special degree distributions, while the Erdös–Rényi algorithm strongly tends towards binomial degree distributions^42^. It is therefore very likely to miss them entirely.

**Fig. 6:**
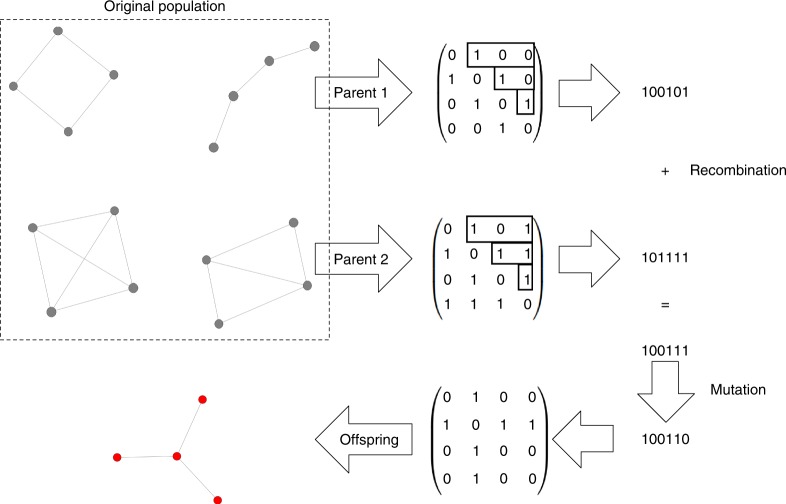
Illustration of the genetic algorithm. A single step of the genetic algorithm for the case of optimizing the fixation probability. Out of a population of graphs, the algorithm chooses two with the highest fixation probability and converts them to the gene code. The gene codes are recombined and mutated and reverted back into an adjacency matrix

We thus propose a heuristic approach based on a genetic algorithm. This algorithm uses a small group of graphs and selects those which optimize a certain property, such as the fixation time, as parents of the group in the next time step. As shown in Fig. 6, the offspring graphs that are generated through recombination and mutation. The procedure is shown in Fig. 6 and can be summarized as follows:

**Parameters:**

*m*: Number of random graphs, here chosen as 120.

*k*: Number of parents per generation, here chosen as 20.

*b*: Number of mutations per individual per time step, here chosen as 1.

*n*: Number of iterations, here chosen as 5000.

1. Generate *m* random Erdös–Rényi graphs.
2. For all graphs, calculate the property to be optimized.
3. Choose the best *k* graphs, based on the property, as parents for the following generation.
4. Generate m new graphs, each with two parents, by recombination. Every link can be inherited from either parent with a probability of 50%. If a resulting graph is not connected, it gets recreated until it is.
5. Mutate these graphs with an average of *b* mutations per individual. A mutation means here that a random link is either eliminated or created. Since every possible mutation is a Bernoulli trial, this results in a Binomial distribution.
6. Repeat steps 2. to 5. *n* times.

Every link has a (low) likelihood of either being mutated or not, independently. Since this is effectively a Bernoulli trial, the resulting sum, the number of mutations per reproduction event, is binomially distributed.

**Fig. 7:**
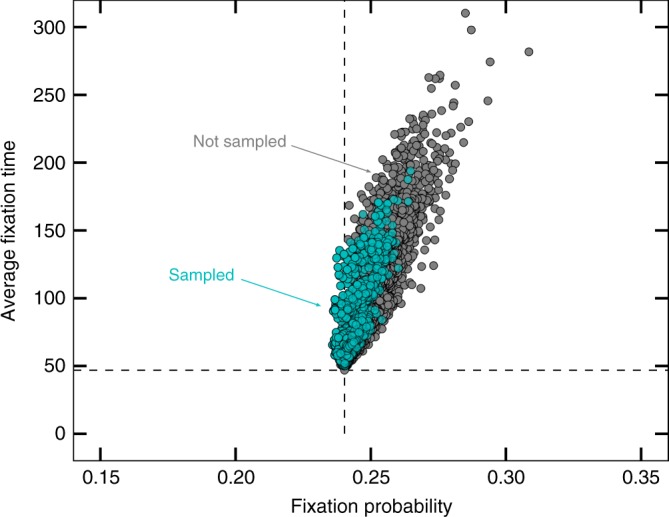
Sampling graphs through the genetic algorithm for size *N* = 8. When we optimize for minimal fixation probability, our genetic algorithm selects only a small subset of graphs, as illustrated here by blue dots plotted on top of the gray dots representing all graphs. Some dots are plotted on top of each other, so that not all sampled graphs are visible. Note that for smaller graphs such as these, we can use fewer graphs for the algorithm and run it for fewer iterations (fitness of mutants *r* = 1.25, *m* = 40, *k* = 5, *b* = 1, and *n* = 1000)

With this approach we only need to look at some relevant graphs for the property we aim to optimize, as opposed to looking at a huge number of graphs. To lessen the likelihood of getting stuck in a specific local minimum, the genetic algorithm can be run multiple times independently and the results can be compared. In addition, we have tested that this genetic algorithm recovers the optima for small graph sizes found by systematically scanning all graphs. Those graphs used by one run of the genetic algorithm to find the graphs with the lowest fixation probability are shown in Fig. 7.

Note that not all graphs are sampled with the same probability: The genetic algorithm randomly generates and deletes links. This means that graphs with a high number of possible representations based on the adjacency matrix are more likely to be generated, while graphs with a low number of possible representations have a lower likelihood to be generated. For example, there is just one representation for the complete graph (every link needs to be present), while there are *N* representations for the star graph (every node can be the center). In general, the more complex a graph is, the more representations it has. It is difficult to asses whether this has a preference towards extremes or towards the center of the probability-time plane. Some extreme graphs, such as the complete graph, and the coupled or generalized star, are quite simple, while the kite-like and detour graphs can be quite complex. But there are also non-extreme graphs that can be simple, such as the line graph. This problem could in principle be remedied by canonically labelling graphs, which would imply that a unique labelling is used for every graph^43^. However, there are still many open mathematical and computational questions concerning such canonical labels^44^ and it is also not clear how to implement mutation and recombination given such labels. Moreover, graphs similar to the complete graph in terms of graph structure usually have similar fixation probabilities^7^, which is a good attribute for a genetic algorithm since it assumes that a graph with a higher fixation probability, for example, is a better primer for the graph with the highest fixation probability. It is unclear so far how true this would be for a potential canonical labeling.

### Reporting Summary

Further information on experimental design is available in the Nature Research Reporting Summary linked to this article.

## Supplementary information

Reporting Summary

Description of Supplementary Movie

Supplementary Information

Supplementary Movie

## Data Availability

The data underlying our figures is available at https://github.com/m-moeller/graph_universe.

## Code availabilty

The simulation code is available at https://github.com/m-moeller/graph_universe.
