# Supplementary material for: Exploring and mapping the universe of evolutionary graphs identifies structural properties affecting fixation probability and time
Source: Commun Biol. 2019 Apr 23;2:137. doi: 10.1038/s42003-019-0374-x (PMC6478964; doi:10.1038/s42003-019-0374-x)
Supplement: Supplementary file 2 — Description of Supplementary Movie [file 42003_2019_374_MOESM2_ESM.pdf]

Description of Additional Supplementary Files

File Name: Supplementary Movie

Description: The supplementary movie is a collection of fixation probability / average fixation time graphs in the case of $N = 8$ for various values of r, keeping the color scheme from Fig. 1 from the main text. It can best be viewed with a browser, but different image viewers also allow to display the figures one by one.
