# Supplementary material for: Exploring and mapping the universe of evolutionary graphs identifies structural properties affecting fixation probability and time
Source: Commun Biol. 2019 Apr 23;2:137. doi: 10.1038/s42003-019-0374-x (PMC6478964; doi:10.1038/s42003-019-0374-x)
Supplement: Supplementary file 3 — Supplementary Information [file 42003_2019_374_MOESM3_ESM.pdf]

1 Update mechanism

Note that all our results in the main text have been derived for a particular update mechanism. Thus, one has to be careful not to overinterpret our results: As Supplementary Figure 1 reveals, our results only hold for selection at birth and random death (Bd), but not for random death and selection at birth (dB).

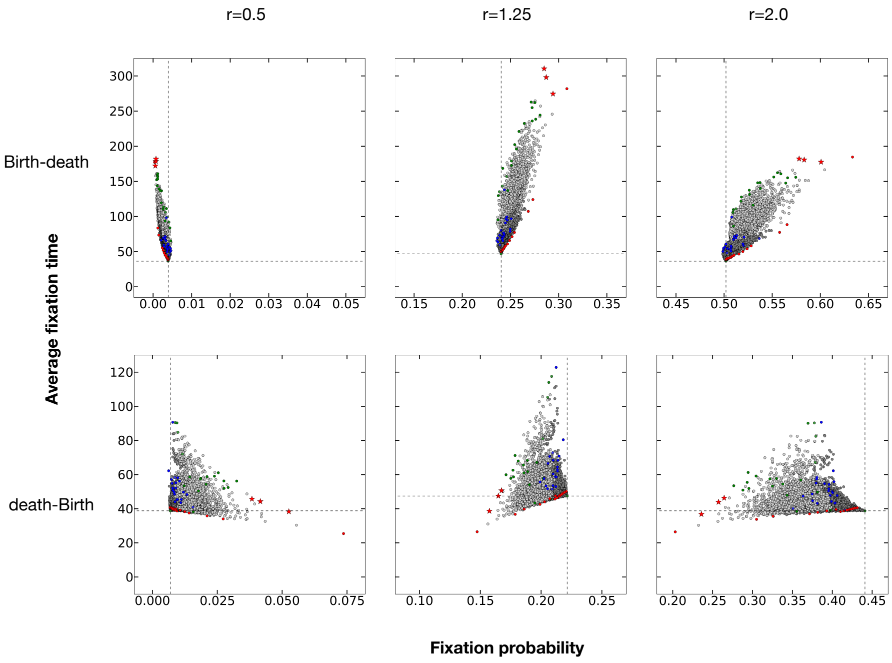

Supplementary Figure 1: **Birth-death vs. death-Birth.** When we switch from global selection for birth and subsequent random local selection for death (Birth-death) to global random selection for death and subsequent local selection for death (death-Birth), the location of graphs in the fixation probability-fixation time plane changes substantially and the special graphs we identified no longer mark the boundaries of the plot.

2 Heat heterogeneity

Given a graph G with N nodes, let the degree of each node be denoted as $d_1, d_2, \cdots, d_N$. The temperature of node i is then defined as $T_i = \sum_{k \in N(i)} \frac{1}{d_k}$, where $N(i)$ is the set of neighbors of node i [1, 2]. Tan et al. define heat heterogeneity as $H_t(G) = \frac{1}{N} \sum_{i=1}^{N} (T_i - \bar{T})^2$, where T_i is the temperature of node $i \in \{1, \cdots, N\}$ and $\bar{T}$ is the average temperature of graph G [2].

Tan et al. found a correlation between heat heterogeneity and fixation probability for Erdős-Rényi (ER) graphs [2]. For graph sizes 4–9 we can study all graphs. Supplementary Table 1 shows the Pearson correlation coefficient (calculated by numpy.corrcoef from the Scientific Python package [3]) between the heat heterogeneity and the fixation probability for all graphs of sizes 4–9 and fitness values $r = 1.25$ and $r = 1.75$. The Pearson correlation coefficient can attain values from -1 to $+1$, -1 reflecting a strong negative correlation and $+1$ a strong positive correlation. The correlations in Table 1 are very high.

**N**	$\mathbf{r = 1.25}$	$\mathbf{r = 1.75}$
4	0.9573	0.9671
5	0.9261	0.9425
6	0.8579	0.8865
7	0.8324	0.8646
8	0.8433	0.8746
9	0.8690	0.8922

Supplementary Table 1: Pearson correlation coefficient between the heat heterogeneity and the fixation probability for graph sizes 4–9. For the computation of these correlations, *all* graphs are included .

For larger graph sizes, we cannot study all graphs. Therefore we use the following procedure to generate graphs: Set a value p for the ER algorithm to be the probability of link connection.

1. Generate an ER graph G with the given p.
2. Extract the degree distribution of G and generate 100 graphs with this degree distribution.
3. Check for uniqueness of these graphs and remove duplicates.
4. If less than 10 unique graphs are left in the set, go back to step 1.
5. Compute the fixation probability and the heat heterogeneity for these graphs.
6. Compute the Pearson correlation coefficient of those.

We repeat this procedure 10 times and compute the average correlation of these 10 trials. The results are displayed in Supplementary Table 2.

N	$\mathbf{r = 1.25}$		$\mathbf{r = 1.75}$	
	$\mathbf{p = 0.5}$	$\mathbf{p = 0.8}$	$\mathbf{p = 0.5}$	$\mathbf{p = 0.8}$
10	0.8743	0.9349	0.8399	0.9688
11	0.8769	0.9677	0.8711	0.9865
12	0.9036	0.9680	0.8921	0.9842
13	0.8873	0.9774	0.9211	0.9832
14	0.9218	0.9746	0.9007	0.9855
15	0.9202	0.9732	0.9135	0.9828
16	0.9049	0.9740	0.9083	0.9890

Supplementary Table 2: Correlation between the heat heterogeneity and the fixation probability for graph sizes 11–16. These correlations are computed from 10 trial runs with 100 graphs each, with an identical degree distribution as one randomly generated ER graph of the given p. Details of this procedure are given in the text and Supplementary Figure 2 show individual data points for a case with $N = 15$.

Supplementary Figure 2 visualizes the correlation of heat heterogeneity and fixation probability for $N = 15$ and $r = 0.5, 1.1, 1.25$, and $r = 2.0$, based on individual data points. These results shows that the degree distribution combined with the heat heterogeneity can predict the fixation probability very well.

References

[1] Lieberman, E., Hauert, C. & Nowak, M. A. Evolutionary dynamics on graphs. *Nature* **433**, 312–316 (2005).

[2] Tan, S. & Lü, J. Characterizing the effect of population heterogeneity on evolutionary dynamics on complex networks. *Scientific Reports* **4**, 5034 (2014).

[3] Scientific Computing Tools for Python. *https://docs.scipy.org/doc/numpy/reference/generated/numpy.corrcoef.html* (accessed February 25th, 2019).

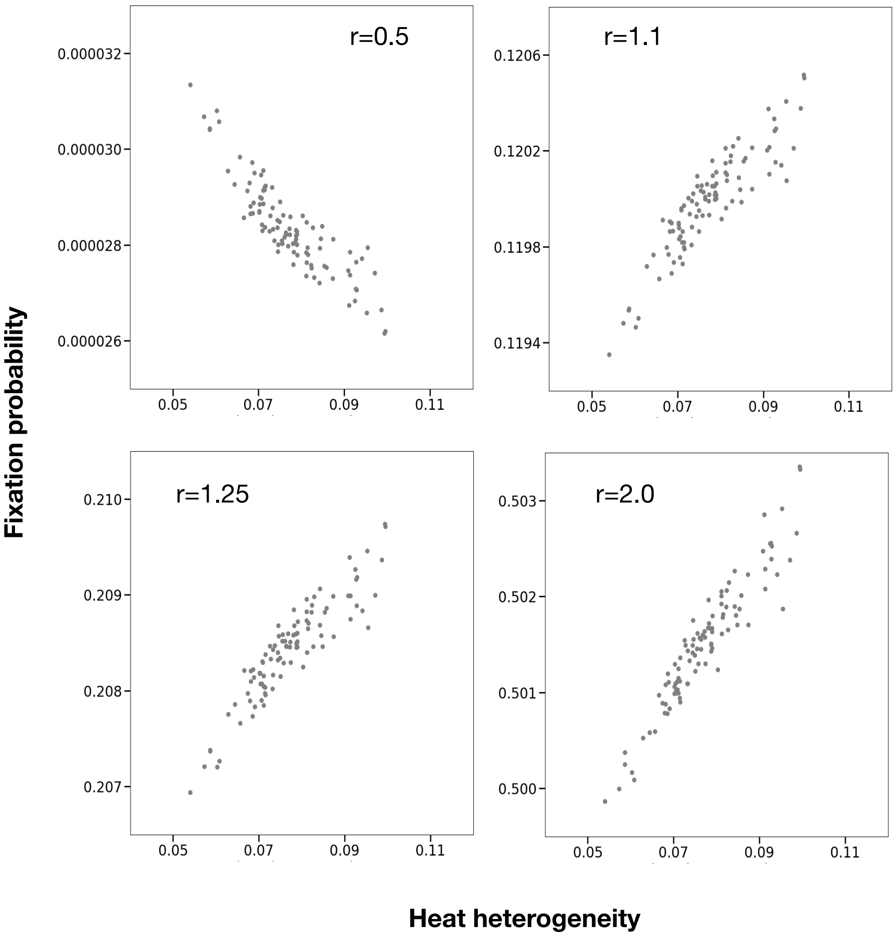

Supplementary Figure 2: **The heat heterogeneity is a good predictor for the fixation probability.** The plots show the correlation between heat heterogeneity and fixation probability for size $N = 15$ and various values of r for a sample of 100 Erdős-Rényi graphs with $p = 0.8$.
