# Supplementary figures and images for: Exploring and mapping the universe of evolutionary graphs identifies structural properties affecting fixation probability and time
Source: Commun Biol. 2019 Apr 23;2:137. doi: 10.1038/s42003-019-0374-x (PMC6478964; doi:10.1038/s42003-019-0374-x)

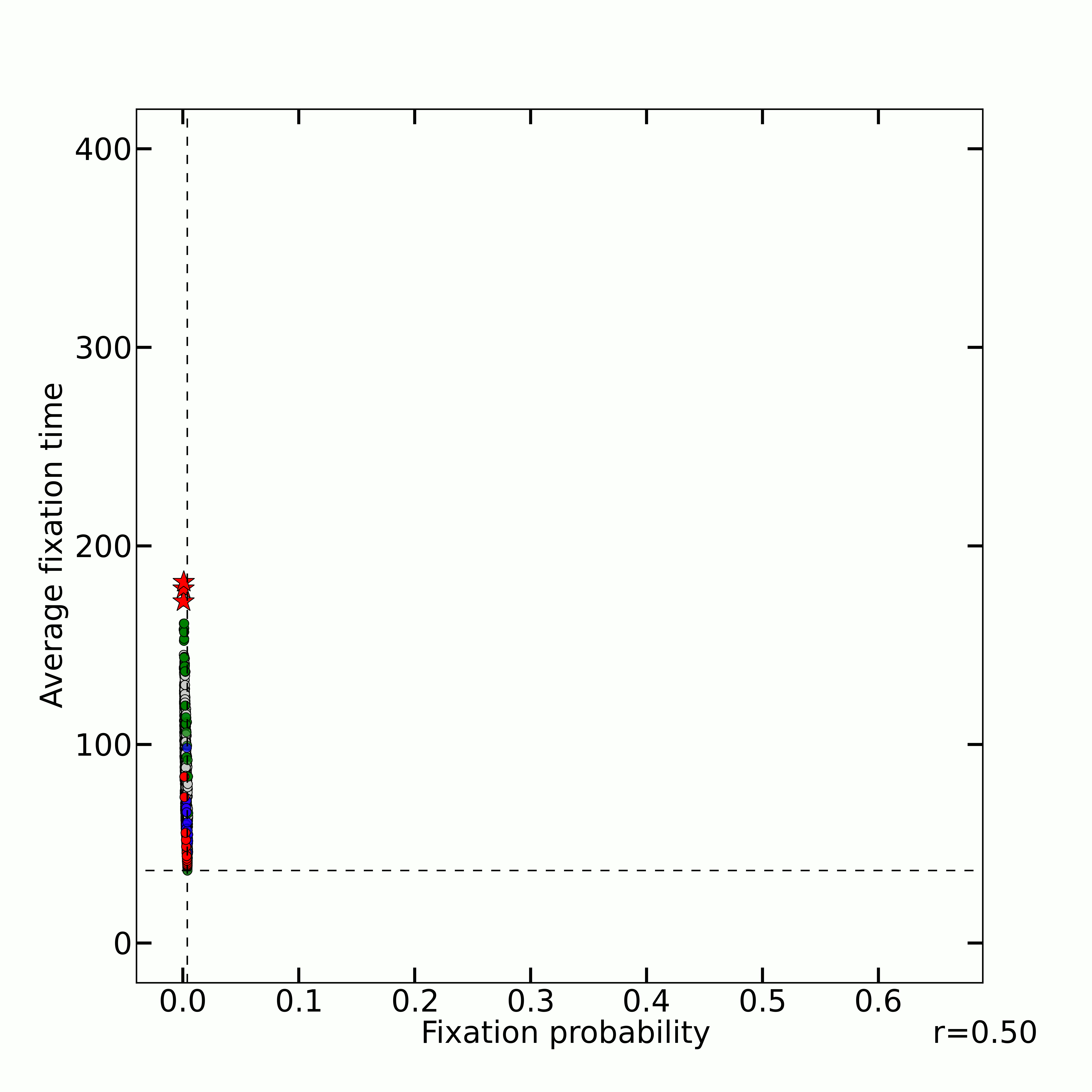

Supplement: Supplementary file 4 — Supplementary Movie [file 42003_2019_374_MOESM4_ESM.gif]
